# Supplementary material for: Cytoskeletal rearrangement precedes nucleolar remodeling during adipogenesis
Source: Commun Biol. 2024 Apr 15;7:458. doi: 10.1038/s42003-024-06153-1 (PMC11018602; doi:10.1038/s42003-024-06153-1)
Supplement: Supplementary file 2 — Supplementary Information [file 42003_2024_6153_MOESM2_ESM.pdf]

Cytoskeletal remodeling defines nucleolar architecture during adipogenesis

Evdokiia Potolitsyna, Sarah Hazell Pickering, Aurélie Bellanger, Thomas Germier, Philippe Collas and Nolwenn Briand

Supplementary information

Supplementary Figures

Supplementary Fig.1 Adipogenic differentiation of hASCs
Supplementary Fig.2 Nuclear remodeling during adipogenesis
Supplementary Fig.3 Protein expression of nuclear and nucleolar markers
Supplementary Fig.4 Nucleolar remodeling during adipogenesis
Supplementary Fig.5 Lineage specificity of nucleolar remodeling
Supplementary Fig.6 Nucleolus morphology during adipogenesis and upon ASC fasting and refeeding
Supplementary Fig.7 Upregulation of ribosomal protein gene expression during adipogenesis
Supplementary Fig.8 Decrease in translation efficiency following adipogenesis
Supplementary Fig.9. Validation of Methylstat treatment.
Supplementary Fig.10 Nucleolus to nucleus volume correlations
Supplementary Fig.11 Gating of BrdU incorporation experiments
Supplementary Fig.12 Representative imaging flow cytometer dataset analysis
Supplementary Fig.13 Uncropped membranes for Supplementary Fig. 2
Supplementary Fig.14 Uncropped membranes for Supplementary Fig. 3
Supplementary Fig.15 Uncropped membranes for Fig. 3g
Supplementary Fig.16 Uncropped membranes for Fig. 4b and Supplementary Fig. 8a
Supplementary Fig.17 Uncropped membranes for Fig. 4d and Supplementary Fig. 8b
Supplementary Fig.18 Uncropped membranes for Fig. 4f and Supplementary Fig. 8c
Supplementary Fig.19 Uncropped membranes for Supplementary Fig. 9

Supplementary Tables

Supplementary Table 1. Antibodies and dilutions
Supplementary Table 2. Software and plugins for image quantification

Supplementary Figures

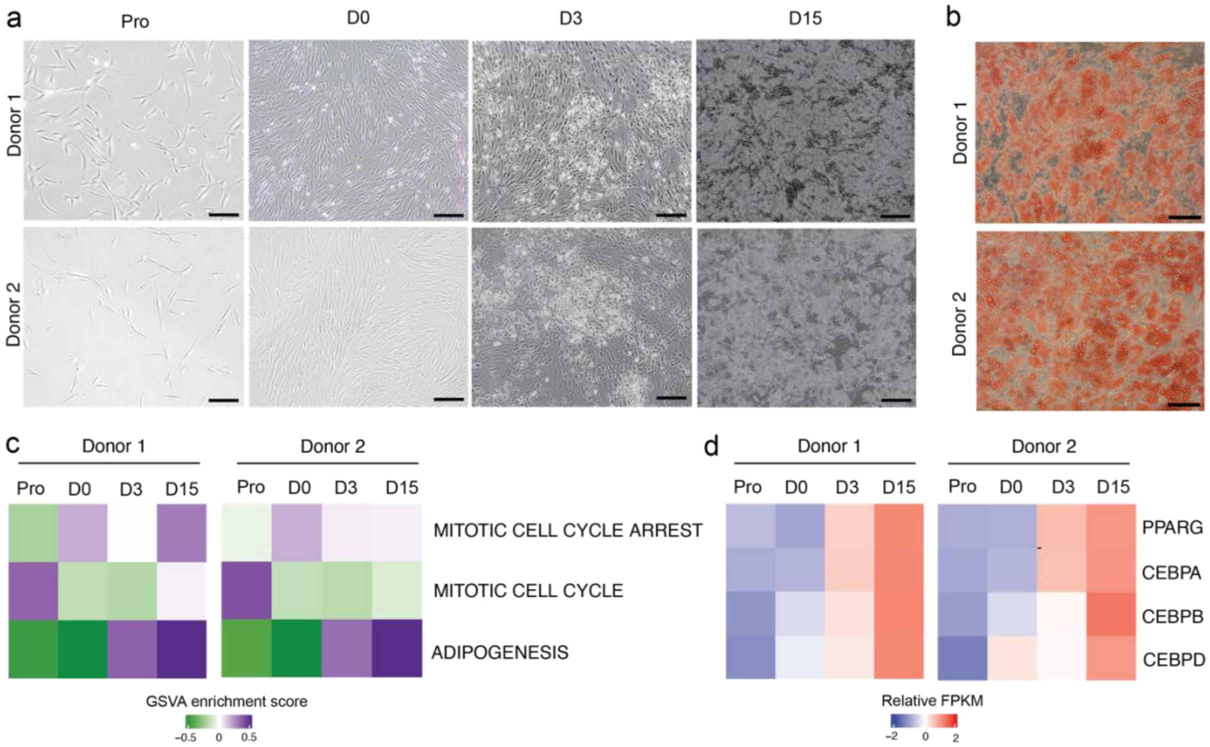

Supplementary Fig.1. Adipogenic differentiation of hASCs. a Phase contrast images of proliferating (Pro), confluent (D0), differentiating (D3) and differentiated (D15) hASCs from two unrelated donors (scale bar: 200 µm). **b** Oil red O staining of neutral lipids at D15 (scale bar: 100 µm) **c** Heatmap representation of the Gene Set Variation Analysis (GSVA) enrichment score for average gene expression of genes pertaining to "Mitotic Cell Cycle Arrest" and "Mitotic cell cycle" GO terms and to "Hallmark Adipogenesis". **d** Heatmap representation of normalized FPKM for key adipogenic transcription factors.

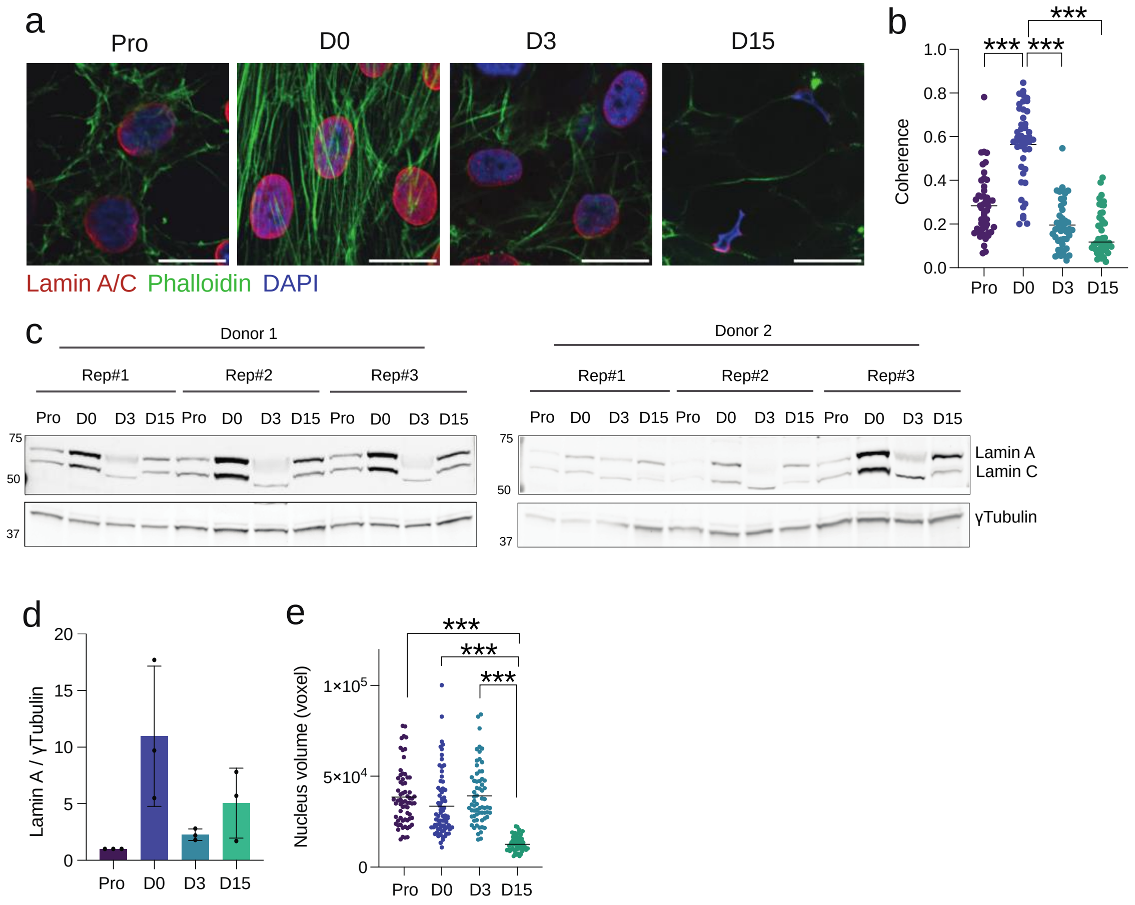

Supplementary Fig. 2. Nuclear remodeling during adipogenesis. a Immunofluorescence of lamin A/C, Phalloidin and DAPI stainings in differentiating ASCs from a second independent donor (donor 2; scale bar: 20 µm). **b** Relative cytoskeleton coherence measured from phalloidin signal (donor 2; ***p < 0.00001 vs D0, two-way ANOVA with Dunnett's multiple comparison test; n ≥ 5 fields of 2500 μm^2). **c** Western blot analysis of Lamin A and Lamin C expression in differentiating ASCs from Donor 1 (left panel) and Donor 2 (right panel). γTubulin is shown as a loading control. **d** Lamin A signals normalized to γTubulin signals quantified from Western blots (Donor 2; non-significant, one-way ANOVA with Holm-Šídák's multiple comparisons test, n = 3 independent experiments). Data are presented as mean ± SD. **e** Nuclear volumes (voxel) measured from DAPI signal (Donor2; ***p < 0.0001 vs D0, one-way ANOVA with Tukey's multiple comparison; n ≥ 30 cells per time-point from independent 3 experiments).

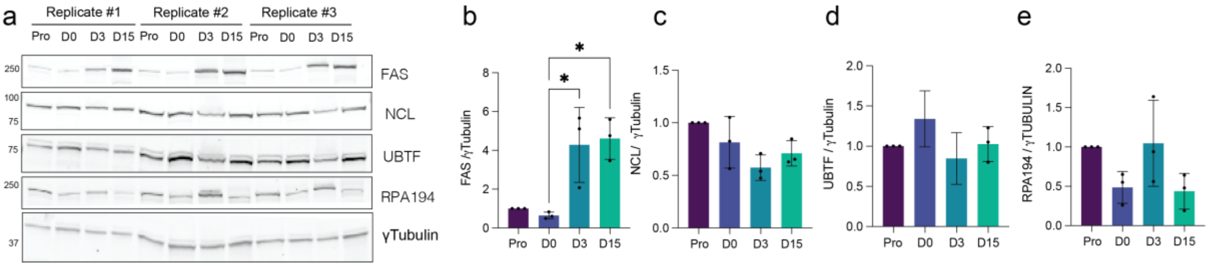

Supplementary Fig. 3. Protein expression of nuclear and nucleolar markers. a Western blot analysis of fatty acid synthase (FAS), nucleolin (NCL), UBTF and RNA Pol1 (RPA194) expression in differentiating ASCs (Donor 1). γTubulin is shown as a loading control, and FAS as a differentiation control. **b**,**c,d,e** Quantification of FAS, NCL, UBTF and RPA194 protein levels normalized to γTubulin in differentiating ASCs (*p < 0.05, One-way ANOVA with Holm-Šídák's multiple comparisons test; n = 3 experiments). Data are presented as mean ± SD.

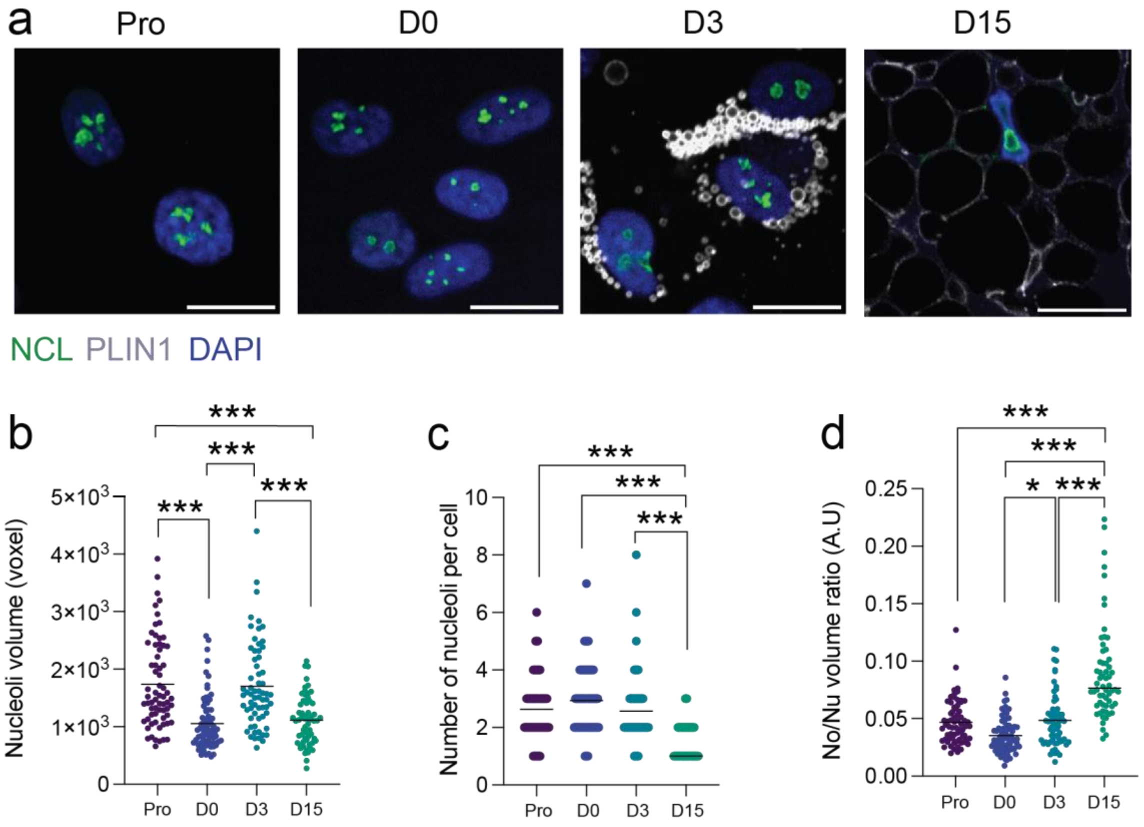

Supplementary Fig. 4. Nucleolar remodeling during adipogenesis. a Immunofluorescence of nucleolin (NCL), perilipin1(PLIN1) and DAPI staining in differentiating ASCs (Donor 2; scale bar: 20 µm). **b** Scatter plot of nucleolar volume measured from nucleolin immunostaining (***p < 0.0001, two-way ANOVA with Tuckey's multiple comparison; n ≥ 60 cells per time-point from 3 experiments). **c** Scatter plot of the number of nucleoli per cell (***p < 0.0001, two-way ANOVA with Tukey's multiple comparison test; n ≥ 60 cells per condition from 3 experiments). **d** Scatter plot of nucleolus-to-nucleus volume (No/Nu) ratio (*p < 0.05, ***p < 0.0001 two-way ANOVA with Tukey's multiple comparison; n ≥ 60 cells per condition from 3 experiments).

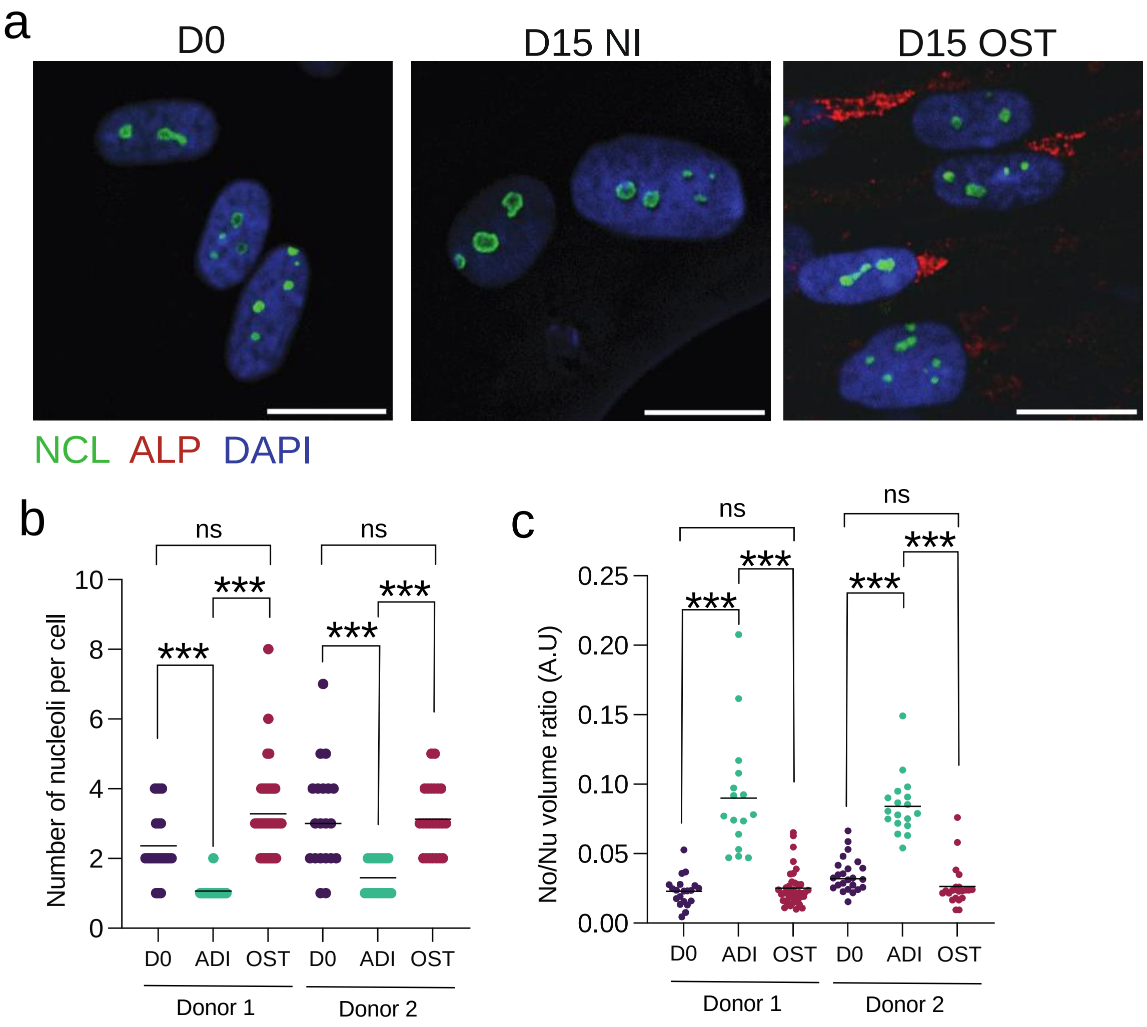

Supplementary Fig. 5. Lineage specificity of nucleolar remodeling. a Immunofluorescence of nucleolin (NCL) and alkaline phosphatase (ALP) and DAPI staining in D0 ASCs, and after 15 days in basal medium (non induced, NI) or osteogenic differentiation medium (OST)(Donor 1; scale bar: 20 µm) **b** Number of nucleoli and **c** nucleolus-to-nucleus (No/Nu) volume ratio measured from nucleolin and DAPI staining, respectively, in D0 ASCs and on D15 of adipogenic (ADI) or osteogenic (OST) differentiation (***$p < 0.0001$, two-way ANOVA with Tukey's multiple comparison test; $n \geq 15$ cells per condition from 3 independent experiments).

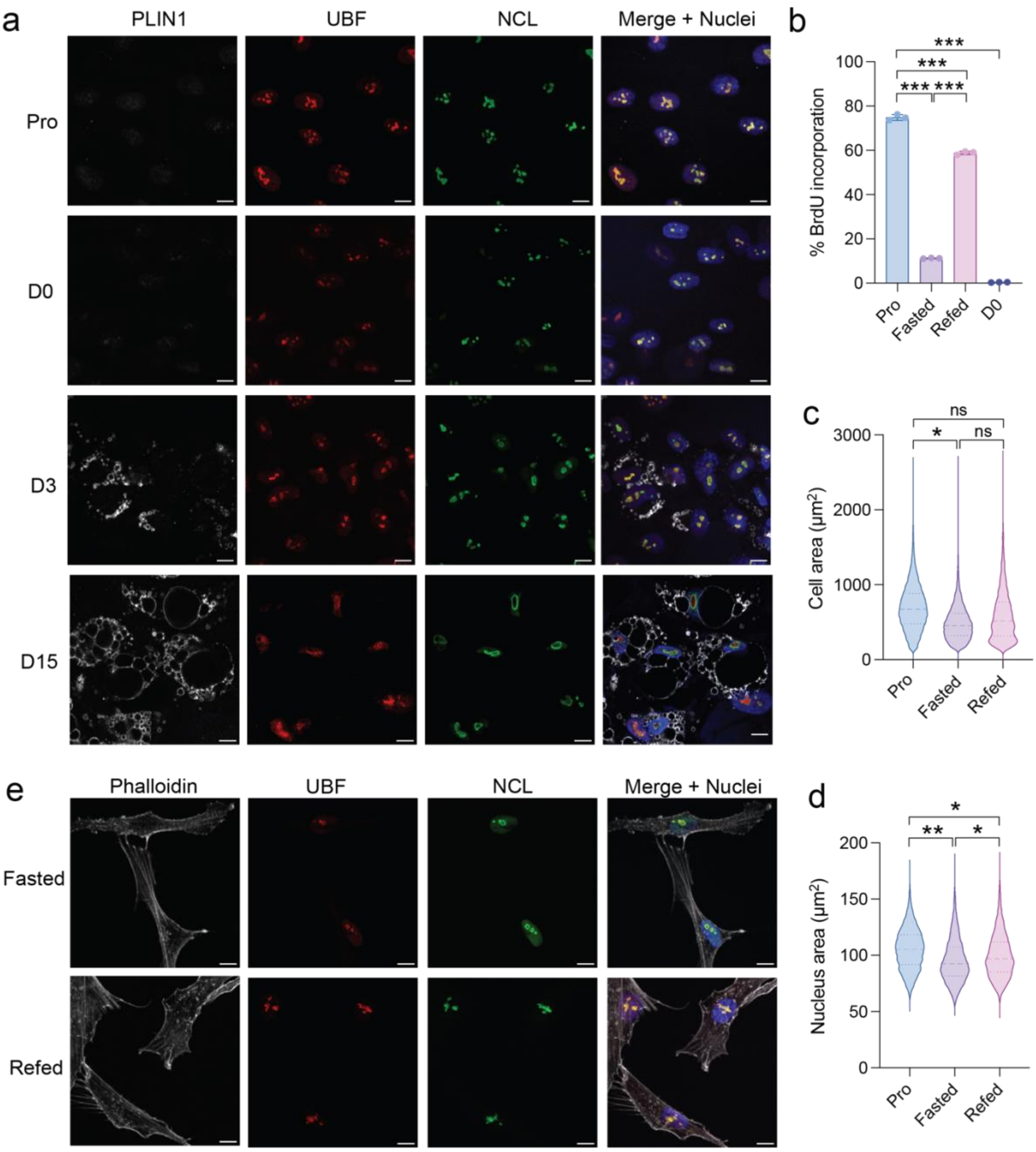

Supplementary Fig. 6. Nucleolus morphology during adipogenesis and upon ASC fasting and refeeding. a Immunofluorescence analysis of UBTF, nucleolin (NCL), perilipin1(PLIN1), and DAPI staining in differentiating ASCs (scale bar: 10 µm). **b** Percentage of BrdU positive cells after 18h BrdU incubation in Pro Fasted Refed and D0 conditions (***p < 0.0001 one-way ANOVA with Holm-Šídák's multiple comparisons test, n = 3). Data are presented as mean ± SD. **c** Cell area and **d** nuclear area measured from imaging flow cytometry data (*p < 0.05, **p < 0.01, ns: non-significant repeated mesures two-way ANOVA with post-hoc pairwise t-test with Holmberg adjustment for multiple testing, n=3). **e** Immunofluorescence analysis of UBTF, nucleolin (NCL) and Phalloidin and DAPI stainings in proliferating ASCs after 24 h of fasting (fasted) followed by a 24-h culture in Pro medium (scale bar: 10 µm).

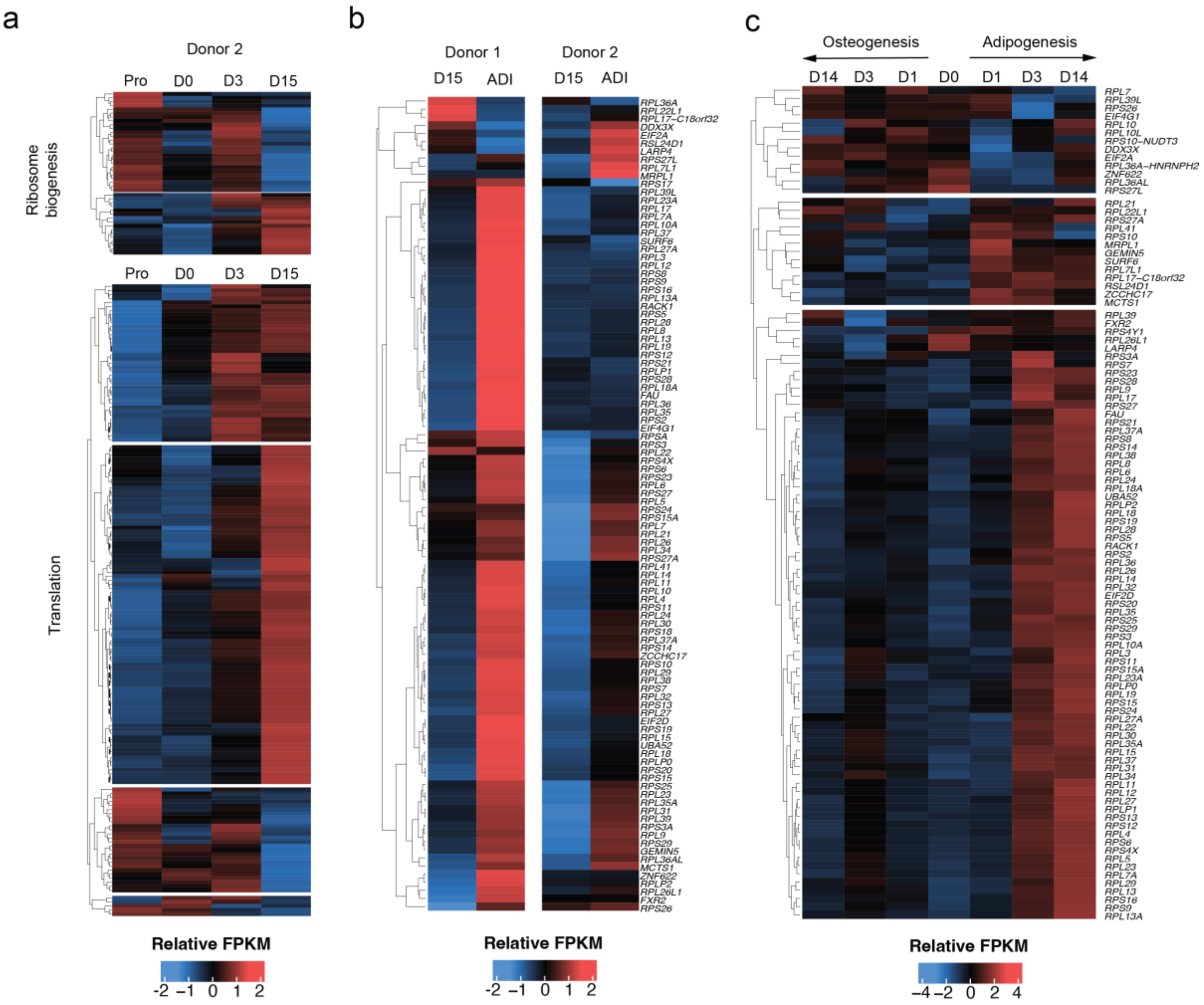

Supplementary Fig. 7. Upregulation of ribosomal protein gene expression during adipogenesis. **a** Heatmap representation of relative gene expression (log2 FPKM) for genes pertaining to the ribosome biogenesis (GO:0042254) and translation (GO:0006412) gene ontology during adipogenesis (Donor 2; $p < 0.01$, eBayes method, limma package). **b** Heatmap representation of relative gene expression (log2 FPKM) for genes pertaining to the Cytosolic ribosome (GO:0022626) gene ontology in D15 cell population vs isolated mature adipocytes (ADI) ($p < 0.01$, eBayes method, limma package). **c** Heatmap representation of relative gene expression (log2 FPKM) for genes pertaining to the Cytosolic ribosome (GO:0022626) gene ontology during adipogenic and osteogenic differentiation of ASCs using an independent RNAseq dataset (Rauch et al. 2019).

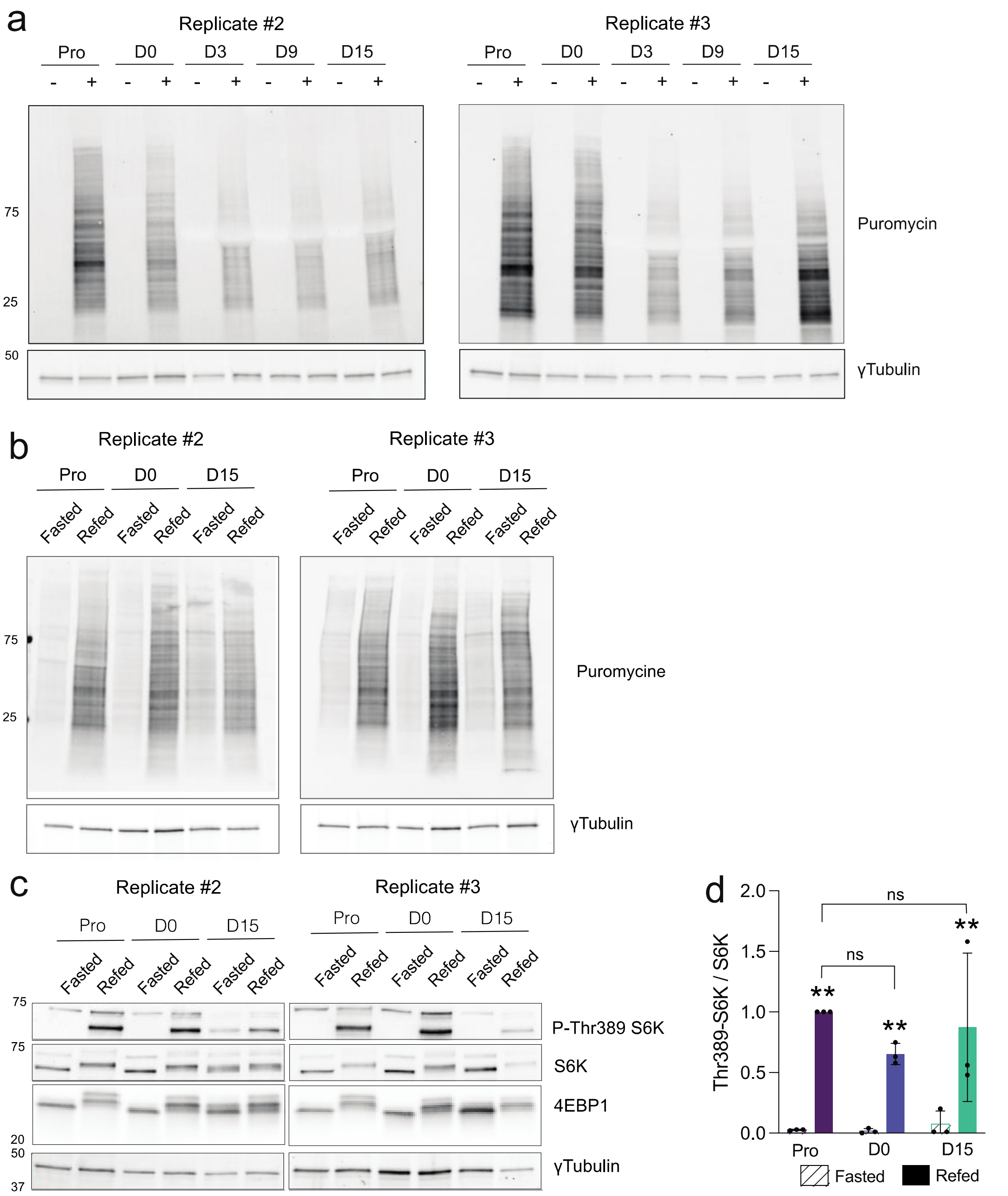

Supplementary Fig. 8. Decrease in translation efficiency following adipogenesis. a SUnSET analysis of protein synthesis rates during adipogenesis with (+) or without (-) 1µM Puromycin (Donor 1; Replicates #2 and #3). γTubulin is shown as a loading control. **b** SUnSET analysis of protein synthesis rates in fasted and refed conditions during adipogenesis (Donor 1; Replicates #2 and #3). γTubulin is shown as a loading control. **c** Western blot analysis of P-Thr389 S6K, total S6K and 4EBP1 in fasted and refed conditions during adipogenesis (Donor 1; Replicates #2 and #3). γTubulin is shown as a loading control. **d** P-Thr389 S6K signals normalized to total S6K signals, quantified from Western blots (Donor 1; **$p < 0.01$, two-way ANOVA with Sidák's multiple comparisons test; $n = 3$). Data are presented as mean ± SD.

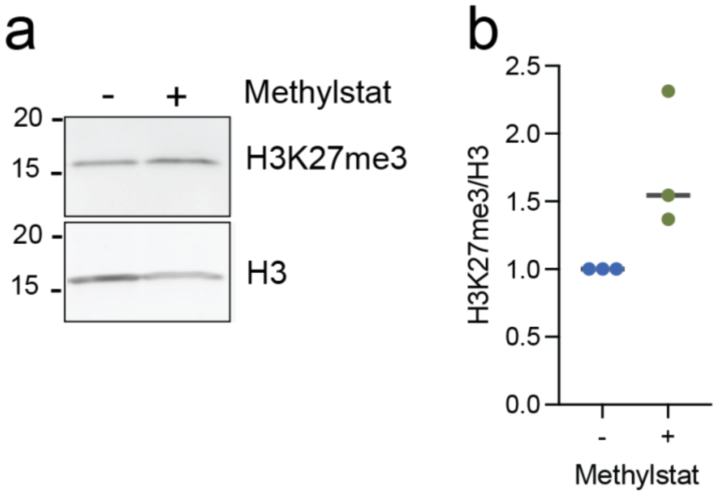

Supplementary Fig. 9. Validation of Methylstat treatment. a Western blot analysis of H3K27me3 levels in control (-) and Methylstat treated (+) proliferating cells. H3 is shown as a loading control. **b** Quantification of relative H3K27me3 levels normalized to total H3 levels.

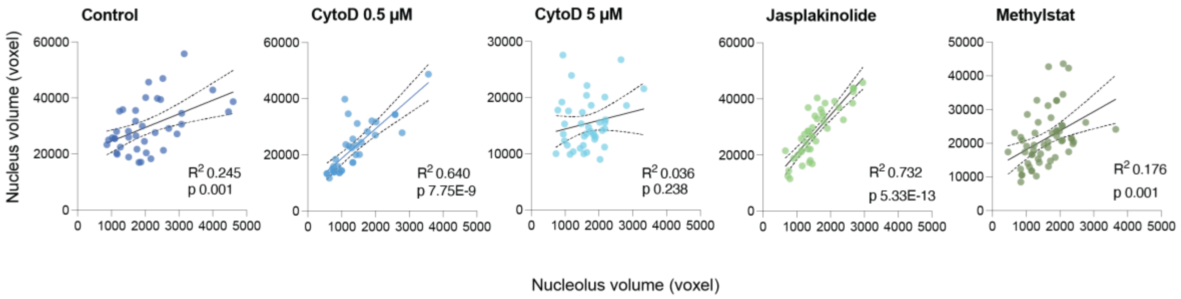

Supplementary Fig. 10. Nucleolus to nucleus volume correlations. Scatter plots of nucleolar volume vs. nuclear volume and linear correlation with 95% confidence intervals bands for control ASCs (Donor 1) and after cytochalasin D (CytoD 0.5 µM and 5 µM) , Jasplakinolide 2 µM or Methylstat 1 µM treatments (simple linear regression; slope R^2 and p-values are shown on the graphs).

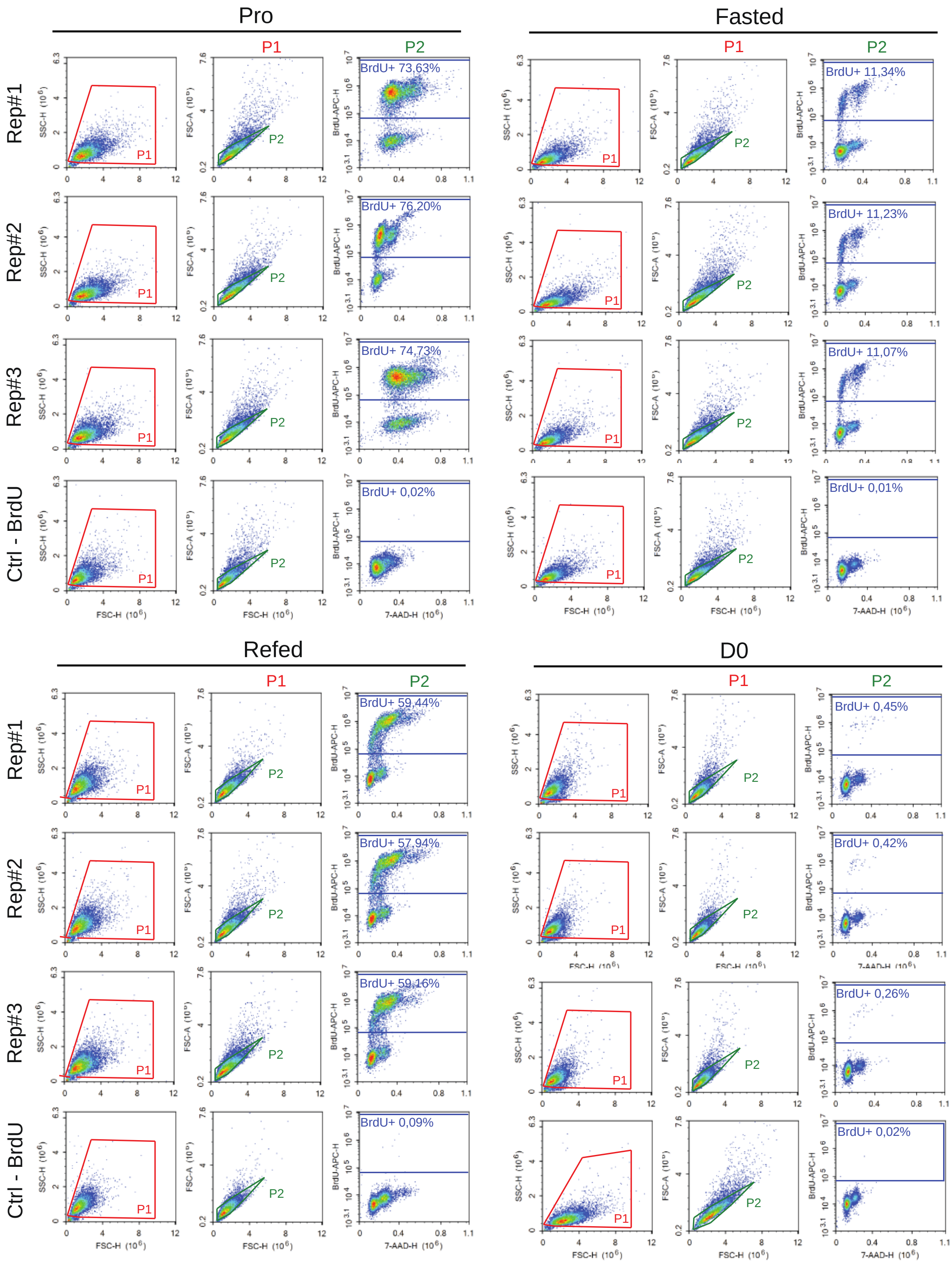

Supplementary Fig. 11. Gating of BrdU incorporation experiments

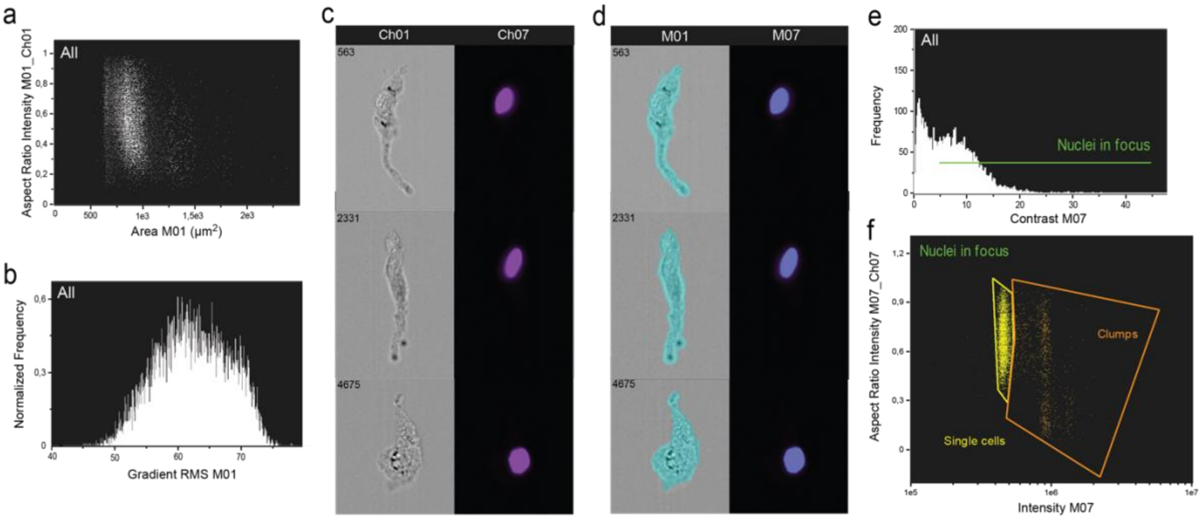

Supplementary Fig. 12. Representative imaging flow cytometer dataset analysis. **a** Scatter plot of area *vs* aspect ratio intensity and **b** histogram of gradient RMS distribution of the total cell population (All) after data acquisition. **c** Representative images of brightfield (Ch01) and Hoechst (Ch07) channels and **d** masked versions of the same images. **e** Histogram of nuclear mask (M07) contrast for the gating of in focus nuclei. **f** Gating of single cells based on nuclear staining intensity from the "Nuclei in focus" population.

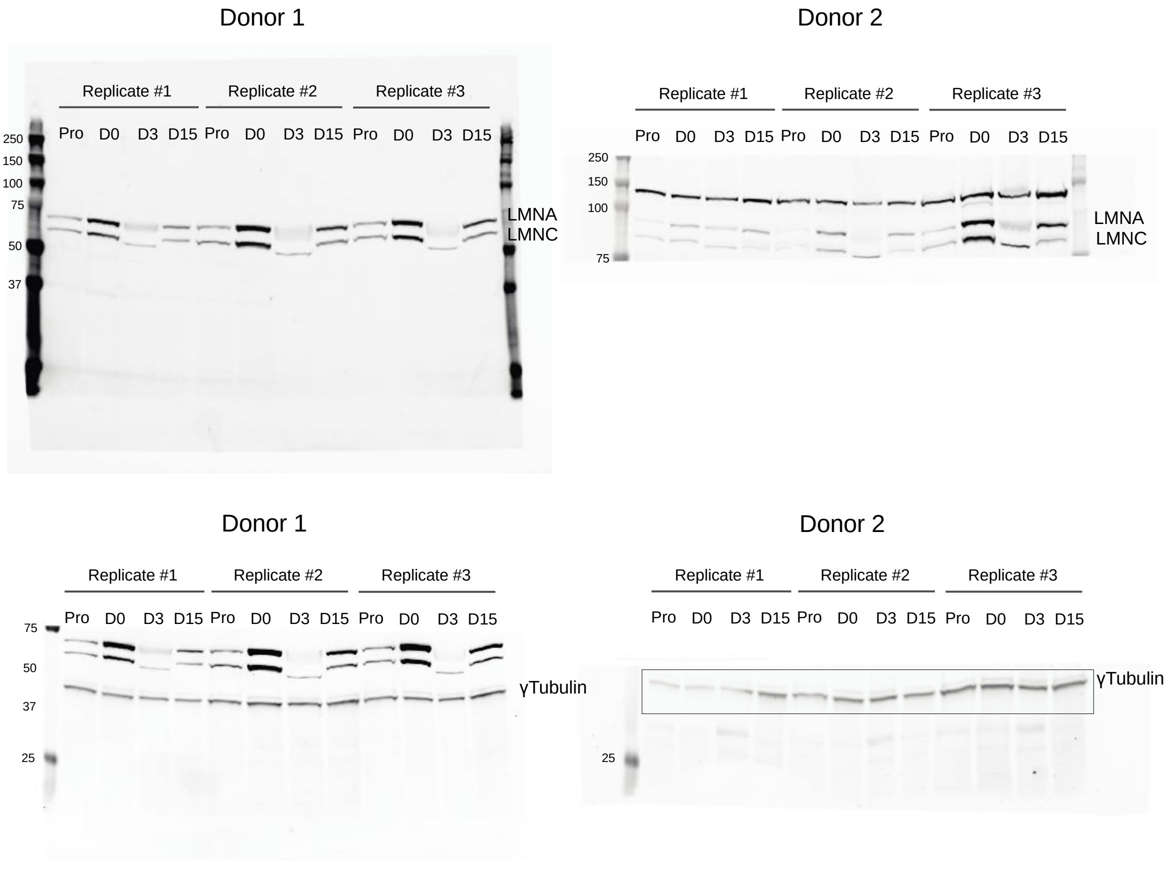

Supplementary Fig. 13. Uncropped membranes for Supplementary Fig. 2.

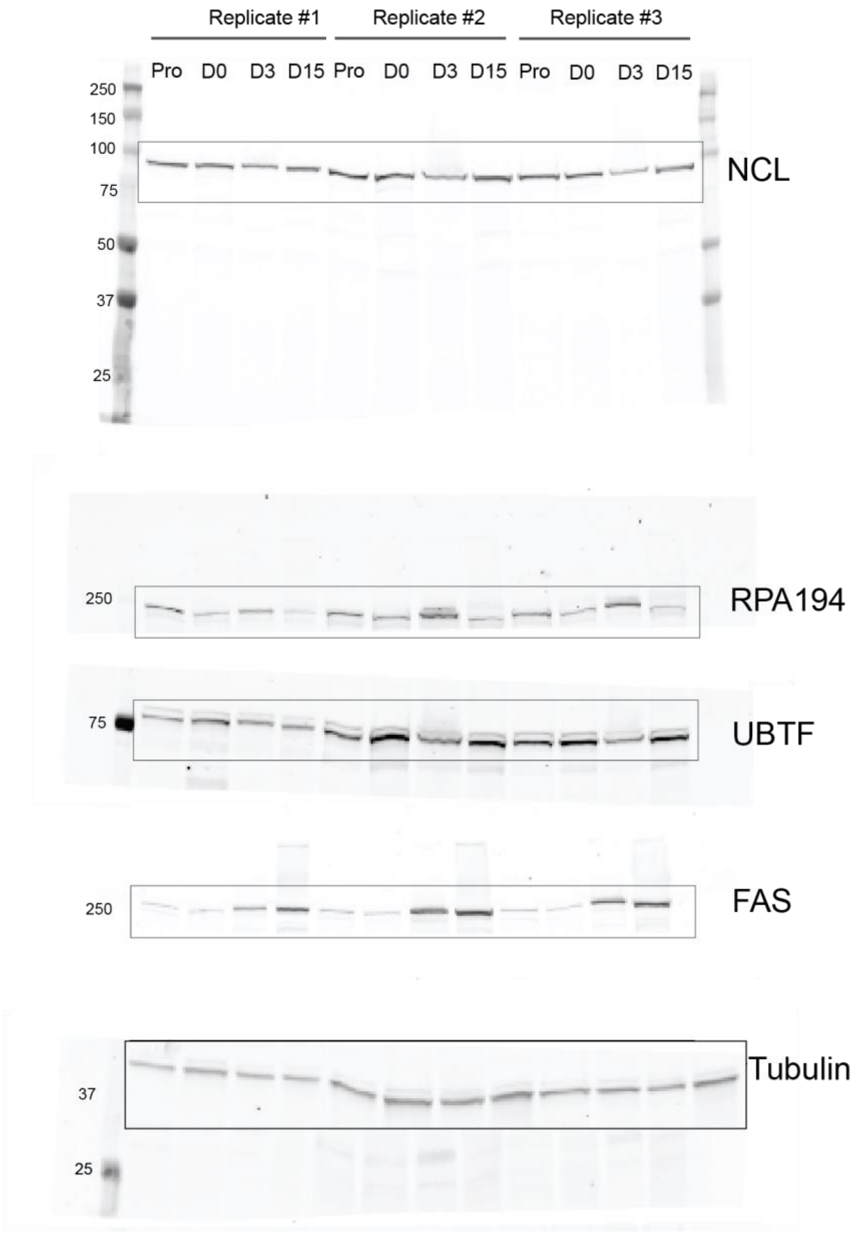

Supplementary Fig. 14. Uncropped membranes for Supplementary Fig. 3

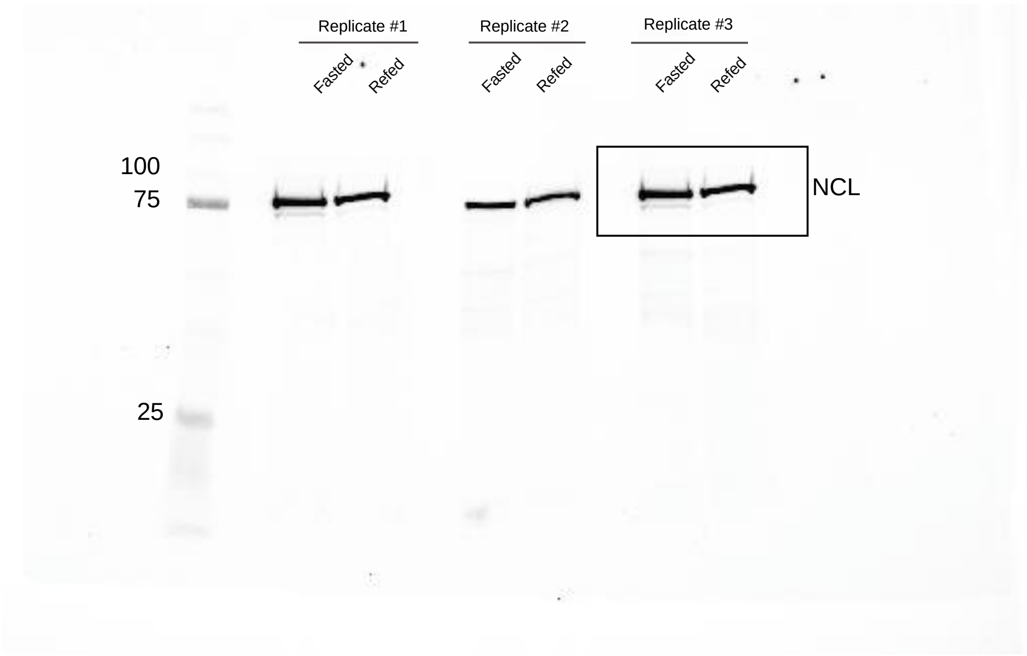

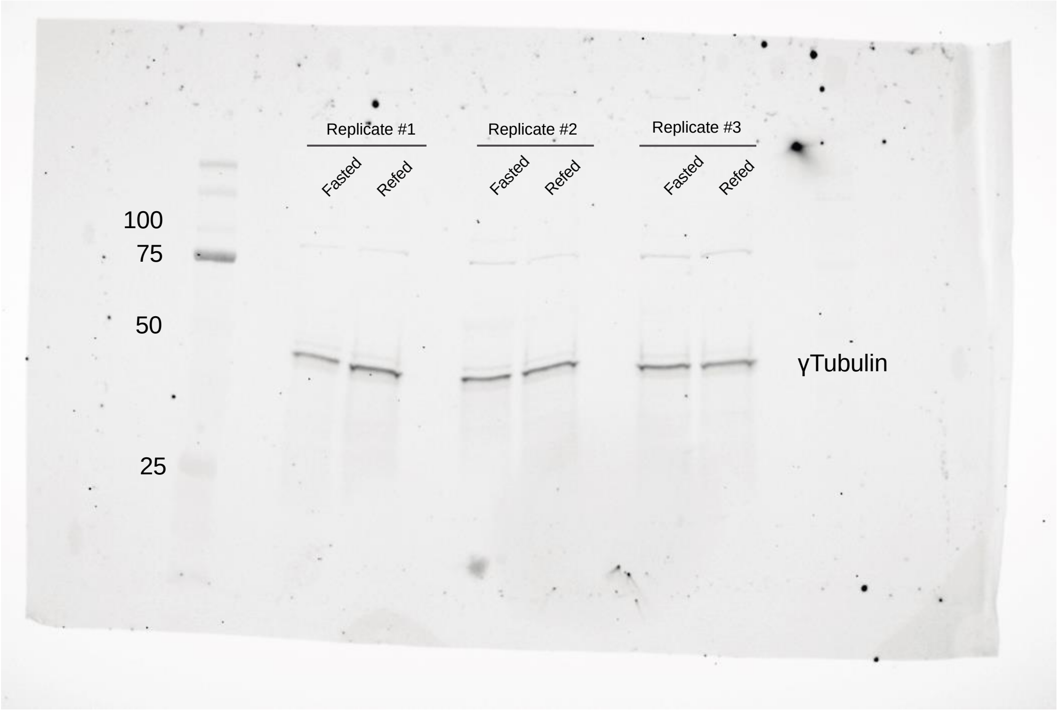

Supplementary Fig. 15. Uncropped membranes for Fig. 3h

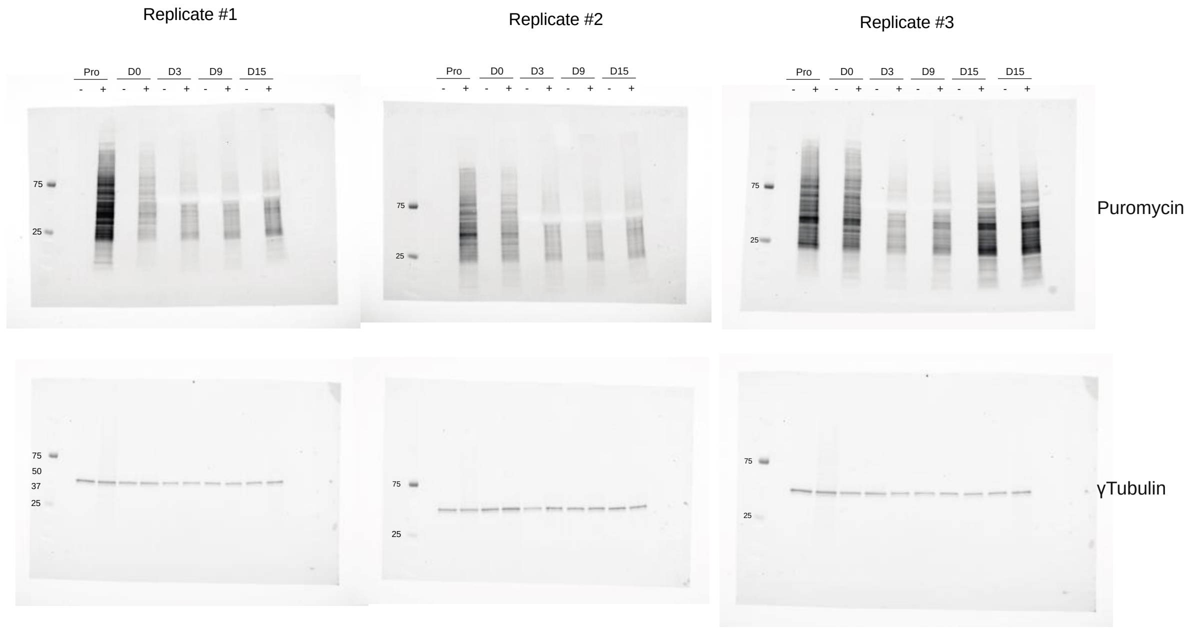

Supplementary Fig. 16. Uncropped membranes for Fig. 4b and Supplementary Fig. 8a

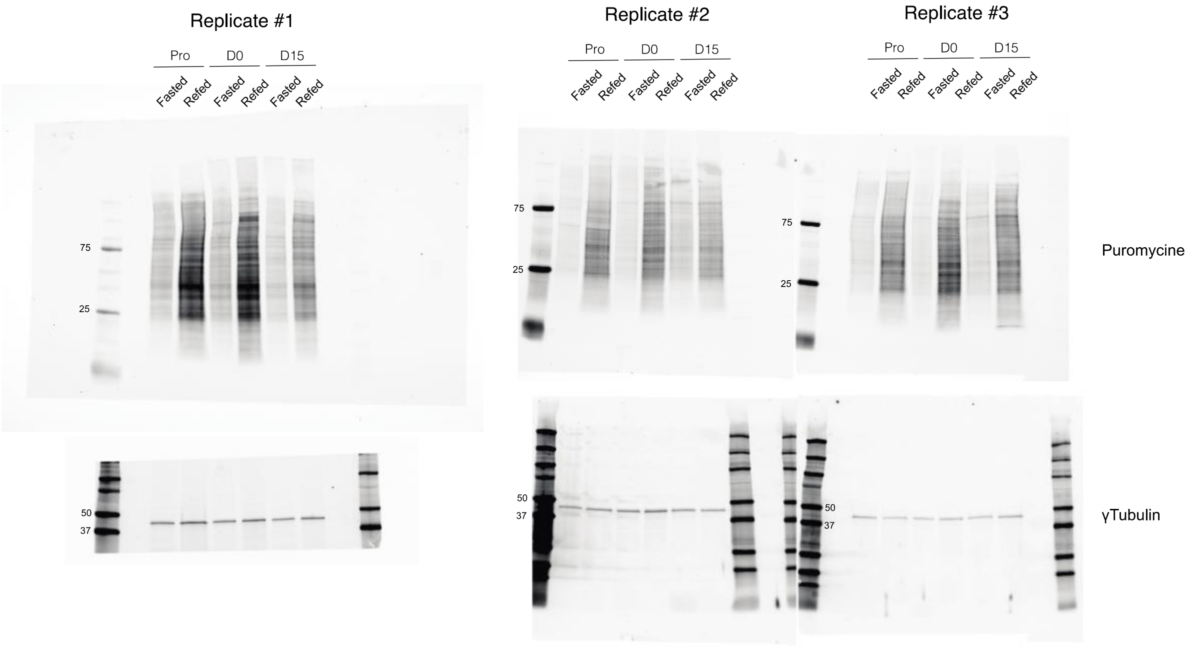

Supplementary Fig. 17. Uncropped membranes for Fig. 4d and Supplementary Fig. 8b

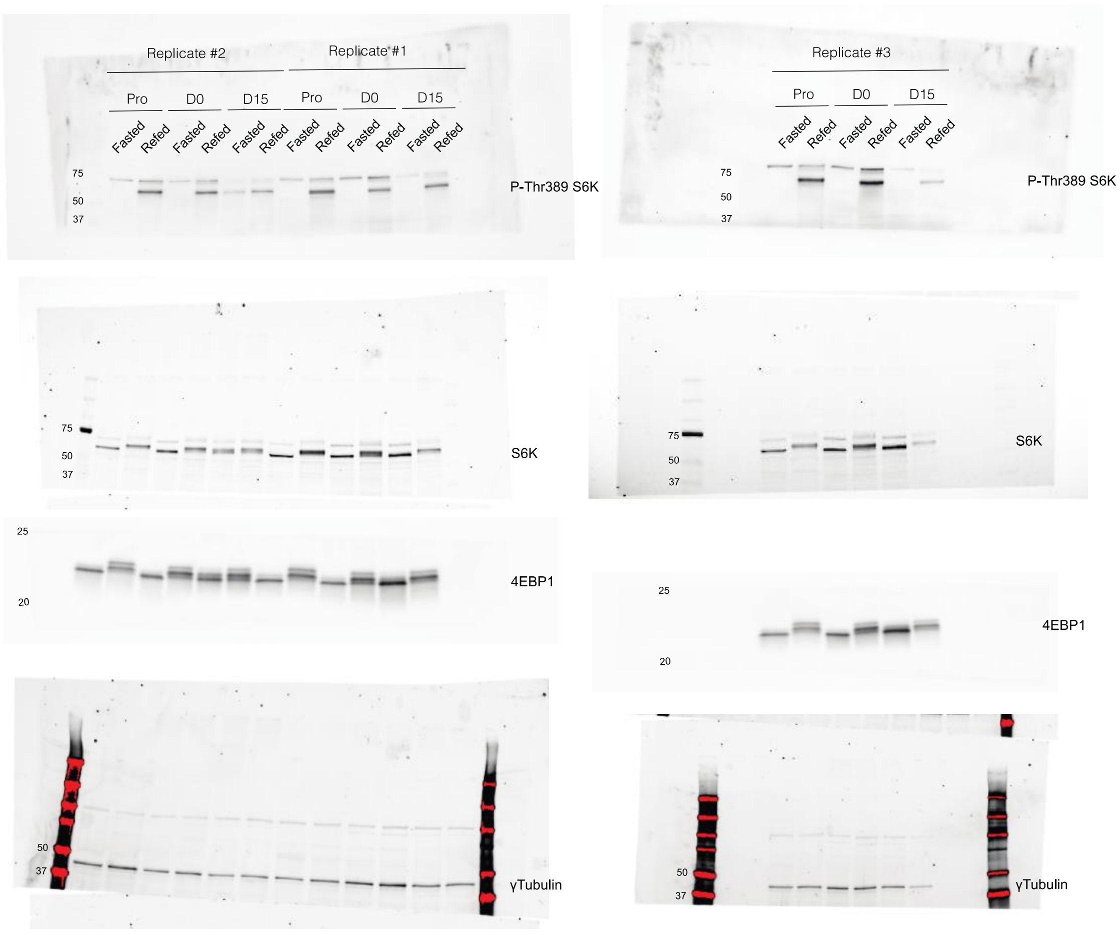

Supplementary Fig.18 Uncropped membranes for Fig. 4f and Supplementary Fig. 8c

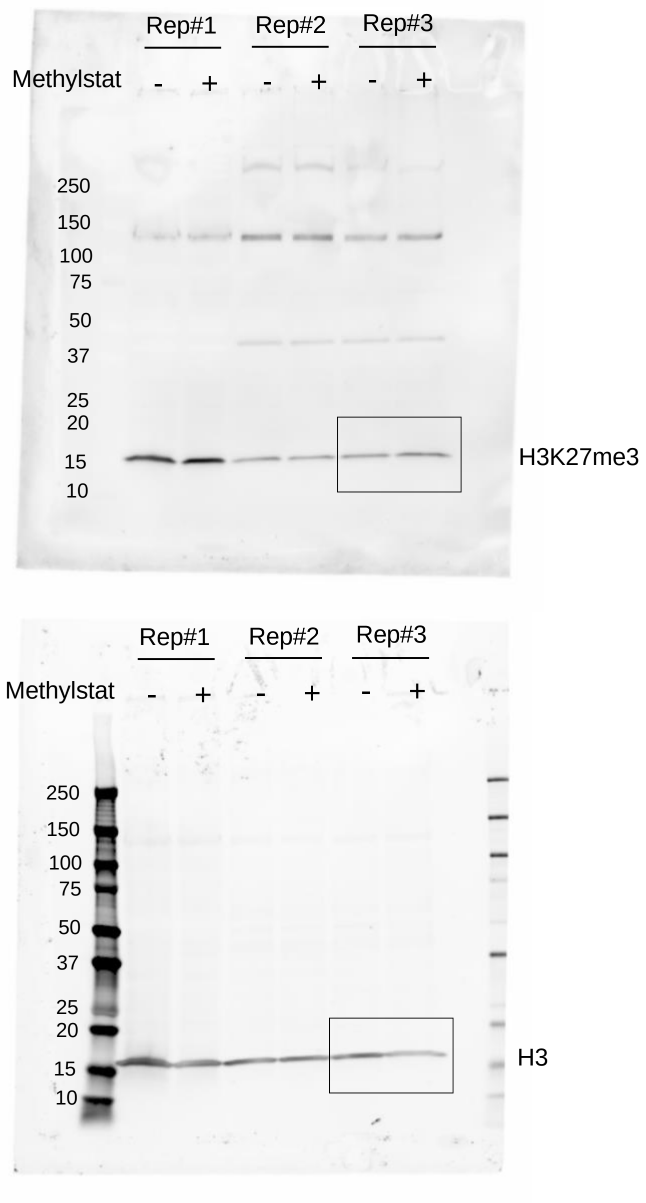

Supplementary Fig.19 Uncropped membranes for Supplementary Fig. 9

Supplementary Table

	Reference	Western Blot	Immunofluorescence
**Lamin A/C**	Santa Cruz, sc-7292	1/500	1/1000
**Nucleolin**	Abcam, ab22758	1/1000	1/1000
**Perilipin1**	Progen, GP29	1/1000	1/500
**Nucleophosmin**	Santa Cruz, sc-32256	-	1/500
**RPA194**	Santa Cruz, sc-48385	1/1000	1/500
**UBTF**	Santa Cruz, sc-13125	1/500	1/200
**FAS**	Santa Cruz, sc-48357	1/1000	-
**β-Actin**	Proteintech, 66009_1_Ig	-	1/1000
**Puromycin**	Millipore, MABE343	1/10000	-
**P-Thr389 P70S6K**	Cell signaling, #9234	1/1000	-
**P70S6K**	Cell signaling, #9202	1/1000	-
**4EBP1**	Cell signaling, #9452S	1/1000	-
**Alkaline Phosphatase**	Abcam, ab17272	-	1/250
**H3K27me3**	Sigma, 07-449	1/1000	
**H3**	Santa Cruz, Sc-8654	1/500	
**γTubulin**	Sigma, T5326	1/10000	-

Table S1. Antibodies and dilutions.

Plugin/Software	Measurement	Image type
**MiNa/ImageJ**	Actin cytoskeleton branch lengths and total volume	full z-stack
**NucleusJ/ImageJ**	Nuclear shape	full z-stack
**OrientationJ/ImageJ**	Actin cytoskeleton coherence	single slice
**MorphoLibJ/ImageJ**	Morphological Segmentation and measure of lipid droplet area	single slice
**Nemo**	Nuclear and nucleolar volumes, number of nucleoli per cell	full z-stack
**Imaris**	Number of UBF foci	full z-stack

Table S2. Software and plugins for image quantification
