# Supplementary material for: Cytoskeletal rearrangement precedes nucleolar remodeling during adipogenesis
Source: Commun Biol. 2024 Apr 15;7:458. doi: 10.1038/s42003-024-06153-1 (PMC11018602; doi:10.1038/s42003-024-06153-1)
Supplement: Supplementary file 5 — Reporting Summary [file 42003_2024_6153_MOESM5_ESM.pdf]

nature portfolio

Corresponding author(s): Nolwenn Briand

Last updated by author(s): 2024 03 18

Reporting Summary

Nature Portfolio wishes to improve the reproducibility of the work that we publish. This form provides structure for consistency and transparency in reporting. For further information on Nature Portfolio policies, see our Editorial Policies and the Editorial Policy Checklist.

Statistics

For all statistical analyses, confirm that the following items are present in the figure legend, table legend, main text, or Methods section.

n/a	Confirmed	
☐	☒	The exact sample size (*n*) for each experimental group/condition, given as a discrete number and unit of measurement
☐	☒	A statement on whether measurements were taken from distinct samples or whether the same sample was measured repeatedly
☐	☒	The statistical test(s) used AND whether they are one- or two-sided<br>*Only common tests should be described solely by name; describe more complex techniques in the Methods section.*
☐	☒	A description of all covariates tested
☐	☒	A description of any assumptions or corrections, such as tests of normality and adjustment for multiple comparisons
☐	☒	A full description of the statistical parameters including central tendency (e.g. means) or other basic estimates (e.g. regression coefficient) AND variation (e.g. standard deviation) or associated estimates of uncertainty (e.g. confidence intervals)
☐	☒	For null hypothesis testing, the test statistic (e.g. *F*, *t*, *r*) with confidence intervals, effect sizes, degrees of freedom and *P* value noted<br>*Give P values as exact values whenever suitable.*
☒	☐	For Bayesian analysis, information on the choice of priors and Markov chain Monte Carlo settings
☒	☐	For hierarchical and complex designs, identification of the appropriate level for tests and full reporting of outcomes
☒	☐	Estimates of effect sizes (e.g. Cohen's *d*, Pearson's *r*), indicating how they were calculated

Our web collection on statistics for biologists contains articles on many of the points above.

Software and code

Policy information about availability of computer code

Data collection	Images were taken with a Dragonfly microscope (Oxford Instruments, EMCCD camera, 60 x 1.4 NA objective, 4-µm stack).
Data analysis	RNAseq was analyzed using hisat2 2.1.0, subread 2.0.1, R 4.2.0 (limma 3.52.4, biomart 2.52.0, complexheatmap 2.12.0)<br>Images were quantified with ImageJ (National Institutes of Health) with OrientationJ plugin (http://bigwww.epfl.ch/demo/orientation/), and with NEMO software (Ianucelli et al. 2010).<br>Intensities of blots were quantified with Image Lab 6.0 (BioRad)<br>Plots and statistical analysis were done with GraphPad Prism 8.4.2.<br>Heatmaps were generated in R using ComplexHeatmap v2.16.0, GSEABase v1.62.0 and GSVA v1.48.3

For manuscripts utilizing custom algorithms or software that are central to the research but not yet described in published literature, software must be made available to editors and reviewers. We strongly encourage code deposition in a community repository (e.g. GitHub). See the Nature Portfolio guidelines for submitting code & software for further information.

Data

Policy information about availability of data

All manuscripts must include a data availability statement. This statement should provide the following information, where applicable:

- Accession codes, unique identifiers, or web links for publicly available datasets
- A description of any restrictions on data availability
- For clinical datasets or third party data, please ensure that the statement adheres to our policy

RNA-seq data are available from Gene Expression Omnibus (GEO) accession GSE176020 and GSE227819

Human research participants

Policy information about studies involving human research participants and Sex and Gender in Research.

Reporting on sex and gender	N/A
Population characteristics	N/A
Recruitment	N/A
Ethics oversight	N/A

Note that full information on the approval of the study protocol must also be provided in the manuscript.

Field-specific reporting

Please select the one below that is the best fit for your research. If you are not sure, read the appropriate sections before making your selection.

☒ Life sciences ☐ Behavioural & social sciences ☐ Ecological, evolutionary & environmental sciences

For a reference copy of the document with all sections, see nature.com/documents/nr-reporting-summary-flat.pdf

Life sciences study design

All studies must disclose on these points even when the disclosure is negative.

Sample size	No sample size calculation was performed. Significance was reached by performing 3 independent biological replicates.
Data exclusions	No data were excluded from analysis.
Replication	Experiments were performed in biological triplicates, and in two independent human cell populations to exclude donor specific effects.
Randomization	Treatment conditions were randomly assigned to paralely seeded differentiation replicates.
Blinding	Blinding was not possible for image analysis, as the cells' differentiation stage can be readily identified.

Reporting for specific materials, systems and methods

We require information from authors about some types of materials, experimental systems and methods used in many studies. Here, indicate whether each material, system or method listed is relevant to your study. If you are not sure if a list item applies to your research, read the appropriate section before selecting a response.

Materials & experimental systems

n/a	Involved in the study
☐	☒ Antibodies
☒	☐ Eukaryotic cell lines
☒	☐ Palaeontology and archaeology
☒	☐ Animals and other organisms
☒	☐ Clinical data
☒	☐ Dual use research of concern

Methods

n/a	Involved in the study
☒	☐ ChIP-seq
☐	☒ Flow cytometry
☒	☐ MRI-based neuroimaging

Antibodies

Antibodies used

Lamin A/C, Santa Cruz, sc-7292, Lot#G2312
Nucleolin, Abcam, ab22758, Lot#GR3361334-1
Perilipin1, Progen, GP29, Lot#406070b
Nucleophosmin, Santa Cruz, sc-32256, Lot#E0614
RPA194, Santa Cruz, sc-48385, Lot#C1720
UBTF, Santa Cruz, sc-13125, Lot#D1919
FAS, Santa Cruz, sc-48357, Lot#E3017
Puromycin, Millipore, MABE343, Lot#3305878
P-Thr389 P70S6K, Cell signaling, #9234, Lot#25
P70S6K, Cell signaling, #9202, Lot#20
4EBP1, Cell signaling, #9452S, Lot#12
Alkaline Phosphatase (anti-bone ALP), ab17272 clone 0.G.2
H3K27me3, Upstate, Sigma 07-449, Lot#1999681
H3, Santa Cruz Sc-8654, lot#E090
γTubulin, Sigma, T5326, Lot# 080M4865
B-Actin, Proteintech, 66009_1_Ig, Lot #10021789

Validation

Lamin A/C Santa Cruz, sc-7292 (636) is recommended for detection of Lamin A and Lamin C of mouse, rat and human origin by Western Blotting (starting dilution 1:200, dilution range 1:100-1:1000), immunoprecipitation [1-2 µg per 100-500 µg of total protein (1 ml of cell lysate)] and immunofluorescence (starting dilution 1:50, dilution range 1:50-1:500)

Nucleolin, Abcam, ab22758 is suitable for WB, IP, IHC-Fr, IHC-P, ICC/IF in Mouse, Rat and Human samples.

Perilipin1, Progen, GP29 is validated for WB and ICC/IF in Mouse, Rat and Human samples.

Nucleophosmin, Santa Cruz, sc-32256 is recommended for detection of B23 of mouse, rat and human origin by Western Blotting (starting dilution 1:200, dilution range 1:100-1:1000), and immunofluorescence (starting dilution 1:50, dilution range 1:50-1:500).

RPA194, Santa Cruz, sc-48385 is recommended for detection of RPA194 of mouse, rat and human origin by Western Blotting (starting dilution 1:100, dilution range 1:100-1:1000), and immunofluorescence (starting dilution 1:50, dilution range 1:50-1:500).

UBTF, Santa Cruz, sc-13125 is recommended for detection of UBF of mouse, rat and human origin by Western Blotting (starting dilution 1:200, dilution range 1:200-1:1,000) and immunofluorescence (starting dilution 1:50, dilution range 1:50-1:500).

FAS, Santa Cruz, sc-48357 is recommended for detection of Fatty Acid Synthase of mouse, rat and human origin by Western Blotting (starting dilution 1:1000, dilution range 1:1000-1:5000), and immunofluorescence (starting dilution 1:50, dilution range 1:50-1:500).

Puromycin, Millipore, MABE343 is validated for western blot in human samples. Monoclonal antibodies to puromycin can be used with standard immunochemical methods to directly monitor translation, a method known as surface sensing of translation (SUnSET).

P-Thr389 P70S6K, Cell signaling, #9234 detects endogenous levels of p70 S6 kinase in Human, Mouse, Rat and Monkey samples only when phosphorylated at Thr389. This antibody also detects p85 S6 kinase when phosphorylated at the analogous site (Thr412).

P70S6K, Cell signaling, #9202 p70 S6 Kinase Antibody detects endogenous levels of total p70 S6 kinase protein in Human, Mouse, Rat and Monkey samples. This antibody also recognizes p85 S6 kinase.

4E-BP1 Cell signaling, #9452S Antibody detects endogenous levels of total 4E-BP1 in Human, Mouse, Rat and Monkey samples, independent of phosphorylation.

Anti-bone Alkaline Phosphatase 0.G.2 (ab17272) specifically recognizes a high molecular weight isoform of alkaline phosphatase of human and cow origin. It has been validated for ICC/IF applications, at a 1/250 dilution with one hour incubation .

H3K27me3 (Upstate,Sigma 07-449) is a highly published Rabbit Polyclonal Antibody against the trimethyl-lysine at residue 27 of human histone H3. This protein A purified antibody is dot blot tested for trimethylated lysine 27 specificity and validated for western blot application.

H3, Santa Cruz Sc-8654 (C16), is a is a goat polyclonal IgG against the C-terminus of Histone H3 of human origin. It is validated for western blot application.

γTubulin, Sigma, T5326 antibody recognizes an epitope located within the N-terminal region of γ-tubulin and reacts with rat, hamster, chicken, human, bovine, canine, Xenopus, mouse γTubulin. It is validated for western blot applications.

B-Actin, Proteintech, 66009_1_Ig antibody immunogen is aa Beta Actin Fusion Protein expressed in E. coli. It reacts with Human, Mouse, Rat, Hamster, Monkey, Dog, Pig, Chicken, Rabbit and Zebrafish B-actin. It is validated for immunofluorescence application (diution range 1:500-1:2000)

Flow Cytometry

Plots

Confirm that:

☒ The axis labels state the marker and fluorochrome used (e.g. CD4-FITC).

☒ The axis scales are clearly visible. Include numbers along axes only for bottom left plot of group (a 'group' is an analysis of identical markers).

☒ All plots are contour plots with outliers or pseudocolor plots.

☒ A numerical value for number of cells or percentage (with statistics) is provided.

Methodology

Sample preparation	Cells were treated for 8h before addition of BrdU (10 µM final concentration) and further incubation for 18h. Controls without BrdU were performed for each conditions. Cells were then processed for BrdU and DNA labelling using an APC BrdU kit (BD pharmingen, BDB552598) following manufacturer's instructions.
Instrument	The flow cytometry experiments were conducted on a NovoCyte (Acea Biosciences Inc.) or an Amnis Imagestream Mk II imaging flow cytometer
Software	Flow cytometry analysis were performed using the Novoexpress (Acea Biosciences Inc.) or the Imagestream IDEAS 6.0 software.
Cell population abundance	N/A, no sorting was performed
Gating strategy	An initial gating was performed to exclude cell debris. Negative controls without BrdU were used to define the gating of BrdU-positive cells.

☒ Tick this box to confirm that a figure exemplifying the gating strategy is provided in the Supplementary Information.
